# Correspondence

**Published:** 1869-03-15

**Authors:** 


### CORRESPONDENCE.

CINCINNATI, OHIO, March 4, 1869.

MY DEAR DR.:—The past two weeks have witnessed the Commencements of all our Ohio Medical Schools. The classes have not fallen far short of former years (since the war.)

The Cleveland Medical College had 123 students. The number composing the graduating class is not known to me. The Charity Hospital Medical College had about eighty students. I do not know the size of the graduating class in this school.

I am informed, however, that it is proposed to remove this school to Worcester, an interior town of this state, where it will be connected with a university. Professor Feristone, who is the life of the school, resides at Worcester.

The class of Starling Medical College, of Columbus, numbers seventy-five or eighty.

The Commencement exercises of the Cincinnati College of Medicine and Surgery, twenty-fifth session, on Longworth street and Central avenue, were held, Feb. 16th., in the Christian church, on Sixth street near Mound, in the presence of a large assembly of ladies and gentlemen.

The exercises were commenced with prayer by Rev. James Black, and consisted of addresses and the conferring of the degree. Rev. M. Lilienthal, D. D., addressed the graduates and conferred the degree on the following persons:

M. L. Amick, Scipio, Indiana; J. H. Anderson, New Haven, Indiana; E. B. M. Browne, Cincinnati; W. E. Caddy, Baltimore; Sam. W. Craig, Cincinnati; H. B. Denman, Piqua; Hugh Ferguson, Maxville; Edwin G.

Keifer, Fairfield; Charles A. Lynd, Cincinnati; F. P. Martin, New Matamoras; D. B. Mozee, Cordova, Kentucky; M. C. Mercer, Fairfield; H. F. McCullough, Livonia, Indiana; A. J. McIntosh, Armstrong, Illinois; T. McFeely, Newport, Kentucky; J. A. McKinnon, Oak Harbor; B. B. Potter, Roundhead; S. Broztman, Yellow Springs; S. M. Royer, Williamsburg, Pennsylvania; T. J. Smith, Wakatomica; J. Q. A. Robbins, Abington, Indiana; J. T. Scott, Rutland, Kentucky; A. M. Seatan, Midway; F. Stubbeman, Cincinnati; J. M. Stutzman, Yellow Springs; F. M. Thomas, Scott; W. F. Wood, New Bremen; W. H Yelton, Bradford, Kentucky; Wilson Hobbs.

This school is in a flourishing condition, having, during the past few years, been imbued with new life. Its Prof. of Surgery, Dr. D. S. Young, is completing his work on Military Surgery. The school has, during the year, lost a valuable member, by the resignation of Prof. Carroll.

The Commencement exercises of the Ohio Medical College for the present term, were held at eleven o'clock, March 1st., in the lecture-room of that institution. Beside the class of graduates, there were a great many visitors, among them a number of ladies. The exercises were opened with prayer by Rev. Mr. Gamble, after which diplomas were given to the students, some seventy in number. The congratulatory address was delivered by Judge Wm. Dickson.

It was well received, upon the whole; but the following remark may be accounted for by the well-known disposition of our countrymen to too often become overawed by a visit to foreign climes:

"A French chemist discovers chloroform, and the inquirng genius of a Scotch physician utilizes it in the discovery of its anæsthetic properties."

This statement is calculated to mislead those who are not informed, into the belief that our countrymen have no claims in that direction.

The following are the graduates receiving diplomas:

S. W. Anderson, Henry J. Abbott, John D. Axline, Chas. F. Basford, William E. Burch, Daniel L. Brown, John H. Bruce, Clinton Brown, Joseph R. Ballard, Simon L. B. Blacke, Samuel L. Beeler, A. L. Chenoweth, George B. Crawford, Robt. H. Culbertson, Perry D. Covington, Lawson Drais, Eber G. Dorr, W. Elijah de Courcy, Jesse O. Davy, S. B. Emerson, John B. Graham, John Ford, Elijah W. Ford, John P. Freeland, Douglass H. Harding, Thomas H. Harrison, P. E. Holland, Henry Haacke, Asa B. Isham, William H. Jones, J. S. Kelsey, John E. Markle, J. B. F. Morgan, Dennis F. Moss, J. W. McGinnis, John Mackay, jr., Thomas C. Moore, John G. McVay, Samuel B. Morgan, Nathan T. Noble, Thomas Orr, George B. Orr, T. S. Potter, Lieutellis L. Porter, L. S. Rice, John E. Rickey, Theodore N. Rafferty, Robert G. Redd, William H. Rogers, Henly C. Rutter, John C. Sloan, Wm. G. Smith, N. W. Spring, Oliver H. Saxton, Beverly W. Sullivan, Edwin J. Thorn, William E. Tucker, Waddy Thompson, George F. Thomin, Will W. Vinnedge, Daniel Wilson, J. Owen Wall, John H. Willard, James M. Wood, Jonathan M. Wright, Holmes T. Wilson, Jeff. D. Young, Martin V. Young.

Prof. M. B. Wright, who has for near a quarter of a century been connected with the school as Prof. of Obs., retires. His usefulness and ability as Prof. and Dean will long be remembered.

The complimentary banquet given by the Faculty of the College to the graduates of the session took place at the Walnut-Street House the previous evening. The occasion was one for social enjoyment and intercourse, and was improved by the graduates and their friends.

This banquet and that of the Cincinnati College (which took place on the evening of their Commencement) were attended almost exclusively by the students and members of the faculties. The high tone characterizing these entertainments do credit to the discernment of the managers.

Last, but not least, came the Ninth Annual Commencement Exercises of the Miami Medical College, held in the College building, on Twelfth street, between Elm and Plum streets, March 2d, in the presence of the Faculty and a large number of ladies and gentlemen, the friends of the graduates and students, and of medical education in this city.

The exercises were conducted by Professor George Mendenhall, Dean of the Faculty, and were opened with prayer by Rev. Dr. Burns.

Prof. Mendenhall introduced the graduates in a body to Right Rev. C. P. McIlvaine, Episcopal Bishop and President of the Board of Trustees, who, after a few pertinent remarks, proceeded to confer the degree upon the following graduates:

O. F. Adams, Frankfort; James Anderson, Versailles, Ind.; J. Bowman, Elora, Ill.; L. B. Bitz, Evansville, Ind.; W. P. Baily, Stanford, Ind.; S. A. Craig, Triadelphia, W. Va.; F. G. Cross, Konoma, Canada; J. W. Cline, Eaton; James W. Dawson, Cincinnati; A. W. Bavis, St. John, N. B.; D. W. Estil, Bluffton, Ind.; F. R. Eakins, Wheelersburg; E. A Eakins, Ironton; E. C. Farquhar, Putnam; W. W. Foley, Indianapolis, Ind.; James F. E. Fanning, Tiffin; H. S. Green, Cardington; Henry Illowy, Cincinnati; Jacob Kindell, Covington; Wade M. Logan, Cincinnati; B. F. Lamb, Richmond, Ind.; V. T. Lindsay, Warsaw, Ky.; L. S. Lambert, Victoria, Ill.; T. M. Lowry, Fort Recovery; D. D. Moore, Spring Valley; W. S. Mendenhall, Zionsville, Ind.; J. Maatin, Caldwell; R. J. Owens, Brookville, Ind.; J. L. Quinn, Eaton; Thomas C. Rogers, Belfast; J. B. Kitchey, Pomeroy; Isaac Redrow, Fayetteville; Hiram Shepard, Newport, Ind.; W. L. Stoneberger, Osborn; W. H. Spilman, Andersonville, Ind.; J. M. Spear, New Vienna; J. H. Smith, Baltimore, Md.; Jas. O. Steele, Robinson, Ill.; S. S. Selvey, Albany, Ind.; S. D. Tomlinson, Bloomingdale, Ind.; Wesley Thompson, Rensalaer, Ind.; T. M. Todp, West Alexander, Penn.; S. B. Sheldon,

Westboro; W. H. Vandusen, Miner's Point, Wis.; Levi Wild, Cincinnati; Geo. A. Way, Graysville.

The Valedictory address of Prof. Clendenin was very good.

The exercises were closed with the apostolic benediction by Dr. Bruns, after which the graduates were invited by the Faculty to the residence of Dr. Richardson, on Eighth street, where an elegant supper was served up and heartily enjoyed by the company, including a number of invited guests, among whom were prominent physicians and non-professional citizens.

The occasion will long be remembered by the numerous friends of the Faculty, and Prof. B. F. Richardson, in particular, whose liberal hospitalities they enjoyed.

At a meeting of the Cincinnati Academy of Medicine, the following officers were elected for 1869.

President, Dr. W. W. Dawson; First Vice President, Dr. W. B. Davis; Second Vice President, Dr. C. S. Muscroft; Secretary, Dr. J. L. Neilson; Treasurer, Dr. S. J. Unzicker; Corresponding Secretary, Dr. E. Stevens; Librarian, Dr. Hiram Smith; Trustees, Drs. J. J. Quinn, R. McIlvaine, and J. P. Walker.

The Commencement exercises of the Ohio Dental College took place last evening, at half past seven o'clock, in the hall of the College. An address was delivered, and the degrees conferred, by Dr. Keely; also, a valedictory address on behalf of the Faculty, by Prof. E. Rives.

It is hoped that the arrangements being made by Prof. P. S. Connor, of this city, chairman of the committee of arrangements for the excursion to the New Orleans meeting of the American Medical Association, will be satisfactory.

Members who attend from the East will be delighted to view the scenery of the Ohio and Mississippi valleys at this (May) season of the year. Besides, after a seventy-two hours' ride by rail the luxury of one of our palatial steamers will be highly appreciated. In this manner the trip will be made within the means of almost any one who desires to go.

Prof. Connor will issue a circular as soon as definite terms are concluded. It is contemplated that the steamer or steamers will make the round trip from Cairo, Louisville or Cincinnati in twelve or fourteen days, furnishing food and lodging while in port. S.

J. ADAMS ALLEN, M. D.:

DEAR DOCTOR:—Having a case in practice I suppose will interest your numerous readers of THE JOURNAL, I report it in brief.

March 1st.—Was called in council to see a patient supposed to have spotted fever. The patient, twenty-eight years of age, presented the well-marked purple patechiæ and ecchymosis of purpura febrilis.

A very peculiar tone of the voice led me to examine the patient very closely, as there was nothing in the purpura to cause alarm. The face was smooth and fair as an ordinary female. But in answer to my question as to whether she had menstruated naturally, she began to cry, and finally requested her attendants to leave us alone. I then proceeded to make a thorough examination of the sexual organs and found the general appearance of the mons veneris natural in appearance except a fullness of the labia on the left side; a rather more than ordinary growth of hair. But a closer inspection revealed a fully developed penis in the place of a female urethra, with its head tied down by the labia above the natural place for the clitoris. On the left side was a fully developed ovary imbedded in the folds of the labia, with no appearance or pretence of a scrotum. The labia and vagina below appeared as in ordinary women until the finger reached in some two inches, when it met a hard, bony substance, which was unresisting, and in shape of the figure 8. Here the sphincter anis and cervixuteri presented themselves closely connected in an osseous state, both openings being sufficient to admit a finger, but separate and distinct from each other; and both unyielding and inelastic. I introduced a womb sound into the uterus one inch, but it seemed hard, and gave such uneasiness to the patient I ceased further examinations.

There are two remarkable features in this case: 1st. The face of this patient was smooth and fair as any ordimary lady. 2d. There was no outside appearance of anus through the perineum, and the only outlet to the rectum was through the vagina.

The patient had always been healthy, never menstruated, nor ever experienced a feeling of passion. And her sex or condition was not known by any one of the family, being an orphan. Very Truly Yours,

L. A. BABCOCK, M. D.

FREEPORT, ILL., March 11, 1869.

